# The Impact of Short-Term Supplementation With Guanidinoacetic Acid and Creatine Versus Creatine Alone on Body Composition Indices in Healthy Men and Women: Creatine-Guanidinoacetic Acid Affects Body Composition

**DOI:** 10.1155/2024/7815807

**Published:** 2024-10-14

**Authors:** Sonja Baltic, David Nedeljkovic, Nikola Todorovic, Laszlo Ratgeber, Jozsef Betlehem, Pongrac Acs, Valdemar Stajer, Sergej M. Ostojic

**Affiliations:** ^1^Applied Bioenergetics Lab, Faculty of Sport and Physical Education, University of Novi Sad, Novi Sad, Serbia; ^2^Faculty of Health Sciences, University of Pecs, Pecs, Hungary; ^3^Department of Nutrition and Public Health, University of Agder, Kristiansand, Norway

**Keywords:** creatine, guanidinoacetic acid, intracellular water

## Abstract

The main objective of this pilot study was to compare the effects of short-term supplementation with a mixture containing creatine and guanidinoacetic acid (GAA) versus creatine alone on body composition indices in men and women. Twenty-three apparently healthy young adults (mean age: 21.4 ± 0.6 years; 10 females) were randomly assigned to receive either a mixture (consisting of 2 g of creatine monohydrate and 2 g of GAA) or an equimolar amount of creatine monohydrate in a pretest–posttest control group experimental crossover design. After the intervention period, participants entered a 2-week washout phase to minimize any residual effects of the treatment. Body composition was assessed using a multifrequency bioelectrical impedance analysis at baseline (preadministration) and at the 7-day follow-up (postadministration). A significant interaction effect was found for extracellular mass (*p*=0.009), with creatine–GAA outperforming creatine in augmenting extracellular mass across the whole sample. In the male subsample, creatine was superior to the mixture in increasing intracellular water (*p*=0.049), whereas the mixture increased extracellular mass, contrasting with the reduction observed with creatine alone (*p*=0.008). No significant differences between interventions were reported in the female subsample (*p* > 0.05), indicating that adding GAA to creatine may produce unique, sex-specific effects on body composition. Further studies are needed to validate our findings across different demographic cohorts and various interventional regimens.

## 1. Introduction

Guanidinoacetic acid (GAA, also known as glycocyamine) is a direct natural biosynthetic precursor of creatine, a key energy-replenishing phosphagen in all mammalian cells. GAA has been recognized as an effective compound in human nutrition for over 70 years [1]. However, recent studies suggest that its capacity to enhance tissue bioenergetics and work performance can be augmented when coadministered with creatine [2–4]. The creatine–GAA mixture likely targets alternative transport channels and significantly improves energy metabolism, addressing tissues that are difficult to reach with conventional creatine interventions [5]. Intriguingly, a preliminary men-only study indicated that combined GAA and creatine resulted in less weight gain compared to creatine administration alone [6], implying the potential effects of the combination on body composition. This could be of particular interest to weight-sensitive populations, where weight gain due to water retention is recognized as a side effect of creatine supplementation [7]. However, no studies have yet compared the effects of GAA and creatine versus creatine alone on various components of the human body while including both sexes. Therefore, the primary aim of this pilot study was to compare the effects of short-term supplementation with creatine–GAA versus creatine alone on body composition indices, as assessed by multifrequency bioimpedance analysis, in young healthy men and women.

## 2. Methods

### 2.1. Study Participants

The study employed a pretest–posttest control group experimental crossover design. Eligibility criteria for participants included the following: age 18–35 years, apparently healthy, nonobese, nonvegetarians, and not having used any creatine-containing supplements or diuretic drugs in the past four weeks. Eligible participants provided voluntary informed written consent, and ethical approval was obtained from the local Institutional Review Board at the University of Novi Sad (#49-09-14/2023-2-SP); the study adhered to the Declaration of Helsinki (seventh revision).

### 2.2. Interventions

During the study, all participants were randomly allocated using a computer-generated list to receive either a creatine–GAA mixture (CreGAAtine, Carnomed, Novi Sad, Serbia) containing 2 g each of creatine monohydrate and GAA for a total of 4 g per day, or an equimolar amount of creatine monohydrate alone (creatine monohydrate, All Stars Fitness Products GmbH, Peißenberg, Germany), in a crossover design. They were instructed to consume the intervention twice daily, before breakfast and dinner, by dissolving the powder in 250 mL of lukewarm water and consuming it immediately. Both interventions were indistinguishable in appearance, texture, and sensory attributes. The treatment spanned 7 days, during which participants were asked to refrain from using any other dietary supplements. The treatment duration was determined based on evidence that creatine-induced weight gain and water retention typically occur within the first week of intervention [8]. In addition, the participants were asked to avoid making significant changes to their diet and physical activity levels during the study. Following the intervention period, participants underwent a 2-week washout phase to mitigate residual treatment effects [9].

### 2.3. Experimental Protocol

All assessments were conducted at two time points: baseline (preadministration) and the 7-day follow-up (postadministration), with laboratory assessments performed between 08:00 and 11:00 following an overnight fast. Participants were instructed to abstain from physical exercise for 12 h and to avoid consuming alcohol, coffee, tea, fizzy drinks, or energy drinks for 24 h before the measurements. Body composition assessment was conducted using a multifrequency bioelectrical impedance analyzer (BioScan 920, Maltron International Ltd, Rayleigh, Essex, UK). All subjects were assessed in a prone position on a nonconductive surface after resting for approximately 10 min, with four electrodes used (tetrapolar method); two electrodes were applied to the hand and two to the foot. A low-level battery current (max 350 mA) was passed through the body, and absolute measurements of impedance, phase angle, resistance, reactance, and capacitance were taken. Multifrequency domains included 5, 50, 100, and 200 kHz, with an impedance range of 5–1100 Ω (resolution: 0.1–1 R), phase range of 1°–30° (resolution: 0.05°), resistance range of 5–1100 R (resolution: 0.1–1 R), and reactance range of 0–580 R (resolution: 0.1 Ω). The recorded parameters included dry weight, fat mass, fat-free mass, body volume, body density, total body potassium, total body calcium, protein mass, mineral mass, glycogen mass, extracellular fluid, extracellular water, intracellular water, total body water, extracellular mass, extracellular solids, interstitial fluid, plasma fluid, body cell mass, and muscle mass. The reported reliability (intraclass correlation coefficients) for a multifrequency bioelectrical impedance analyzer for major outcome variables was > 0.90 [10]; multifrequency body electrical resistance appears to be a reliable indicator of muscle glycogen accumulation in a well-controlled laboratory setting (for a detailed review, see [11]). In addition, weight retention was assessed using the food retention questionnaire [12], with higher scores on this self-reported measure indicating more severe experiences of water retention, bloating, and weight gain. Data collection took place at the Applied Bioenergetics Lab at the University of Novi Sad between April and May 2024.

### 2.4. Statistical Analyses

The minimal sample size (*n* = 20) was determined via power analysis (G^∗^ Power 3.1.9.3, Heinrich Heine Universität Düsseldorf) based on an effect size of 0.35 (considered a small-to-medium effect), an alpha error probability of 0.05, and a power of 0.80; the primary endpoint was the change in total body water from baseline to the 7-day follow-up. This calculation was designed for two interventions and two measurements of study outcomes. Changes in study outcomes within interventions during the trial were compared using paired *t*-tests for normally distributed data and the Wilcoxon signed-rank test for variables not following a normal distribution. For normally distributed data with homogeneous variances, interaction effects (time vs. intervention) were analyzed using a two-way ANOVA with repeated measures. In cases of nonhomogeneous variances, Friedman's test was employed. The significance level was set at *p* ≤ 0.05. Any missing data were excluded from the analyses. All statistical analyses were conducted using SPSS version 24.0 for Mac (IBM SPSS Statistics, Chicago, IL).

## 3. Results

A total of 26 participants (*n* = 26) were enrolled in the study. Three participants were lost to follow-up, while 23 received the designated treatment and were included in the analysis of study outcomes. The baseline characteristics of the participants are summarized in Table 1. No major adverse effects associated with either intervention were reported during the trial, except for a female participant (age 21) who experienced moderate-to-severe nausea after both interventions. Compliance with the intervention was 98.2 ± 5.3% in the creatine–GAA intervention and 97.6 ± 5.9% in the creatine intervention, as calculated based on the number of unused sachets.

Changes in body composition outcomes during the study for two interventions are depicted in Table 2. The consumption of the creatine–GAA mixture resulted in a significant increase in body weight (*p*=0.01), total body water (*p*=0.04), extracellular mass (*p*=0.02), and glycogen content (*p*=0.03) compared to the baseline values across the entire sample. In the female subsample, no significant changes in body composition indices were observed after creatine–GAA intake (*p* > 0.05). However, in the male subsample, the mixture provoked a significant increase in fat-free mass (*p*=0.05), extracellular mass (*p*=0.01), glycogen content (*p*=0.03), extracellular water (*p*=0.01), interstitial fluid (*p* < 0.01), plasma (*p*=0.01), and extracellular fluid (*p*=0.02). On the other hand, creatine supplementation alone induced no significant changes in body composition outcomes across the entire sample, except for a significant increase in total body calcium mass at the 7-day follow-up (*p*=0.05). No changes were observed in the female subsample after creatine intake (*p* > 0.05). In the male subsample, creatine induced a significant increase in body cell mass (*p*=0.04), total body potassium (*p*=0.04), total body calcium (*p*=0.03), total body water (*p*=0.02), intracellular water (*p*=0.02), and extracellular solids (*p*=0.03). In addition, creatine intake resulted in a significant reduction in extracellular mass (*p*=0.05), protein content (*p*=0.01), and mineral mass (*p*=0.01).

A two-way ANOVA with repeated measures revealed no significant interaction effect (time vs. treatment) between interventions for total body water, the primary study outcome, in the entire sample. However, a significant interaction effect was found for extracellular mass (*p*=0.009). The creatine–GAA mixture increased extracellular mass by an average of 1.44%, while creatine alone reduced it by an average of −0.15%. In addition, there was a nonsignificant trend for an interaction effect using Friedman's test for glycogen content (*p*=0.08) and body volume (*p*=0.06), suggesting a likelihood for creatine–GAA to enhance glycogen levels and body volume compared to creatine alone. No significant interaction effects were found between interventions across all body composition biomarkers in the female subsample (*p* > 0.05). Friedman's test revealed a weak nonsignificant evidence for an interaction effect for body weight (*p*=0.07), indicating a possibility for the creatine–GAA blend to outperform creatine in increasing body weight in women. In the male subsample, a significant interaction effect (time vs. treatment) was found for intracellular water (*p*=0.049) and extracellular mass (*p*=0.008). Creatine demonstrated superiority over the mixture in increasing intracellular water, while the mixture increased extracellular mass, contrasting with the reduction observed with creatine alone. In addition, strong nonsignificant trends for an interaction effect were noted for several body composition indices in the male subsample, including extracellular water (*p*=0.084), protein (*p*=0.086), minerals (*p*=0.088), extracellular fluids (*p*=0.106), plasma (*p*=0.080), and interstitial fluid (*p*=0.080). Finally, weight retention scores were not significantly affected by either intervention during the study (*p* > 0.05). The measured raw data for total body capacitance, phase angle, resistance, and reactance during the study are available in Supporting Table 1.

## 4. Discussion

This pilot trial is likely the first to compare the effects of short-term supplementation with the creatine–GAA mixture versus creatine alone on body composition indices in young, healthy men and women. We demonstrated that the two interventions have distinct effects on study outcomes. Notably, the mixture outperformed creatine in augmenting extracellular mass (a proxy for nonmetabolically active tissues, along with extracellular water) and showed a nonsignificant evidence to surpass creatine in elevating glycogen content and body volume in the entire sample. No significant differences between interventions were reported in women, yet creatine was superior to the mixture in increasing intracellular water in men, whereas the mixture increased extracellular mass, contrasting with the reduction observed with creatine alone. Adding GAA to creatine may thus produce unique, sex-specific effects on body composition. Further studies are needed to validate our findings across different cohorts and various interventional regimens.

Creatine offers numerous health and performance benefits for athletes and clinical populations (for a detailed review, see [13]). However, creatine supplementation often leads to weight gain, likely due to water retention, especially during the first week of intervention [8, 14]. As an osmotically active compound, creatine increases intracellular water when absorbed into skeletal muscles from the bloodstream. This water retention can enhance tissue hydration and potentially stimulate muscle growth [15]. Nonetheless, weight gain can be undesirable for individuals in weight-sensitive populations [7]. Several strategies have been proposed to mitigate this initial weight gain, including tapering the dosage or selecting specific creatine forms [16]. Adding GAA to creatine has emerged as an innovative alternative to reduce creatine-induced weight gain due to water retention. A recent study showed that a combined GAA and creatine mixture (3 g of creatine and 1 g of GAA) administered for 4 weeks resulted in less weight gain in healthy men compared to creatine alone [6]. The present study supports these findings, confirming the superiority of creatine over the mixture in increasing intracellular water in men. It also expands the investigation by examining a specific supplementation period and dosage, providing a more comprehensive evaluation of body composition, and including both sexes. In the male subsample, a significant interaction effect was found for intracellular water: creatine increased intracellular water by an average of 3.43%, whereas the creatine–GAA mixture increased it by only 0.72%. Interestingly, a trend for an interaction effect was observed for other hydration indices in men, with the mixture favoring an increase in plasma volume, interstitial fluid, and extracellular water. This suggests a contrasting hydration capacity between the two interventions, with creatine targeting the intracellular compartment and the creatine–GAA mixture targeting the extracellular compartment. Although no clear mechanism explains this phenomenon, the difference in polarity and hydrophilic capacity between creatine and GAA molecules might determine their hydration capacity [17]. Being less hydrophilic compared to creatine, GAA might have a reduced water-bonding capacity. We also found that the creatine–GAA mixture outperformed creatine in augmenting extracellular mass in both the entire sample and the male subsample. Extracellular mass includes bone, cartilage, ligaments, nonmetabolically active tissues, and extracellular water [18]. This supports the hypothesis that the mixture drives the expansion of the extracellular compartment. No significant interaction effect between interventions for changes in intracellular water was demonstrated in women. However, the creatine–GAA blend showed a weak nonsignificant tendency to outperform creatine in increasing body weight, perhaps due to subtle changes in hydration, in women. This suggests potential sex-specific effects of the intervention for hydration outcomes, with creatine–GAA potentially mitigating intracellular water retention in men while promoting it in women. Interestingly, the creatine–GAA mixture tended to slightly surpass creatine in elevating glycogen content in the entire sample (0.98% vs. 0.32%) although the difference remained nonsignificant. This finding could be particularly relevant for individuals interested in expanding glycogen stores to help regulate blood glucose levels. The observed effect may be attributed to the insulinotropic properties of GAA when consumed as a food compound, superior to other amino acid derivatives and guanidines including creatine [19, 20]. Furthermore, creatine increased total body calcium mass, whereas the mixture showed no effect on this component of body composition. This observation is consistent with prior research indicating the beneficial impact of creatine on the bone remodeling process (for a detailed review, see [21]).

Our pilot trial provides novel insights into the physiological impacts of short-term supplementation with a creatine–GAA mixture on body composition indices in both men and women. However, several limitations must be acknowledged when interpreting these findings. The relatively short duration of the intervention restricts our capacity to ascertain the long-term effects of creatine–GAA intake on body composition indices. Furthermore, the study exclusively targeted young healthy adults, introducing uncertainties regarding potential effects on other demographic cohorts, particularly individuals with obesity or other body composition disorders. The sample size was adequate for the overall analysis but insufficient for the sex-specific subanalyses; thus, the sex-specific findings may be influenced by the small sample sizes for both males and females. Future investigations should incorporate more sophisticated techniques for assessing body composition, such as dual-energy X-ray absorptiometry and magnetic resonance imaging, and perhaps incorporate segmental vs. whole-body analysis. In addition, the study did not include a separate group supplemented exclusively with GAA, complicating the identification of each component's individual contribution to the observed effects.

## 5. Conclusion

The addition of GAA to creatine has been shown to mitigate the initial increase in intracellular water induced by creatine alone in healthy men and exhibit a trend toward outperforming creatine in increasing body weight in women. These sex-specific effects on hydration outcomes, as well as other body composition indices, are of considerable interest in human nutrition. However, they necessitate validation through well-designed longitudinal trials across different cohorts.

## Figures and Tables

**Table 1:** Baseline characteristics of the study participants.

*n*	Female	Male	Total
	10	13	23
Age (years)	21.1 ± 0.3	21.6 ± 0.7	21.4 ± 0.6
Weight (kg)	68.9 ± 10.2	85.0 ± 7.5	78.0 ± 11.8
Height (cm)	172.4 ± 8.8	183.8 ± 2.9	178.9 ± 8.4
Body mass index (kg/m^2^)	23.1 ± 2.1	25.1 ± 1.9	24.3 ± 2.2
Fat mass (kg)	15.9 ± 6.0	11.2 ± 3.7	13.3 ± 5.3
Fat-free mass (kg)	52.9 ± 5.7	73.8 ± 4.2	64.7 ± 11.6
Body volume (L)	66.1 ± 10.7	79.7 ± 7.4	73.8 ± 11.2
Body density (g/cm^3^)	1.04 ± 0.02	1.07 ± 0.01	1.06 ± 0.02
Body cell mass (kg)	27.6 ± 3.0	39.6 ± 2.6	34.4 ± 6.7
Extracellular mass (kg)	25.3 ± 2.7	34.2 ± 1.9	30.3 ± 5.0
Muscle mass (kg)	22.3 ± 2.7	36.6 ± 2.3	30.4 ± 7.6
Total body potassium (g)	122.8 ± 14.1	189.3 ± 12.5	160.4 ± 36.1
Dry weight (kg)	68.1 ± 10.2	83.2 ± 7.5	76.6 ± 11.5
Protein (kg)	9.8 ± 1.4	14.9 ± 1.5	12.7 ± 2.9
Mineral (kg)	4.0 ± 0.6	5.2 ± 0.5	4.7 ± 0.8
Total body calcium (kg)	0.97 ± 0.12	1.49 ± 0.09	1.27 ± 0.28
Glycogen (g)	480.4 ± 51.7	670.2 ± 38.5	587.7 ± 105.6
Total body water (L)	38.7 ± 4.0	53.6 ± 3.5	47.1 ± 8.4
Extracellular water (L)	16.9 ± 2.1	21.6 ± 1.1	19.6 ± 2.8
Intracellular water (L)	22.1 ± 4.5	32.0 ± 2.7	27.7 ± 6.1
Interstitial fluid (L)	12.5 ± 6.1	15.9 ± 0.9	14.5 ± 2.1
Plasma fluid (L)	3.6 ± 0.4	4.6 ± 0.2	4.1 ± 0.6
Extracellular solids (L)	4.9 ± 0.6	7.6 ± 0.5	6.4 ± 1.4
Extracellular fluid (L)	17.9 ± 2.3	22.9 ± 1.2	20.7 ± 3.0
Food retention score (points)	2.1 ± 2.4	0.8 ± 2.0	1.4 ± 2.2

*Note:* Values are expressed as mean ± SD.

**Table 2:** Body composition indices after 7-day supplementation with the creatine–GAA mixture and creatine alone.

	Creatine–GAA			Creatine		
	Female	Male	Total	Female	Male	Total
Weight (kg)	69.4 ± 10.4	85.4 ± 7.5	78.4 ± 11.9^∗^	69.0 ± 10.1	85.3 ± 7.6	78.2 ± 11.9
Fat mass (kg)	16.2 ± 7.1	11.1 ± 3.4	13.3 ± 5.6	15.0 ± 4.3	11.1 ± 3.7	12.8 ± 4.4
Fat-free mass (kg)	53.2 ± 4.9	74.4 ± 4.5^∗^	65.2 ± 11.7	53.0 ± 5.3	74.1 ± 4.5	64.9 ± 11.7
Body volume (L)	66.7 ± 11.6	80.0 ± 7.4	74.2 ± 11.4	64.9 ± 8.4	79.7 ± 7.7	73.2 ± 10.9
Body density (g/cm^3^)	1.04 ± 0.03	1.07 ± 0.01	1.06 ± 0.01	1.05 ± 0.01	1.07 ± 0.01	1.06 ± 0.01
Body cell mass (kg)	27.4 ± 2.7	39.8 ± 2.9	34.4 ± 6.8	27.4 ± 2.9	40.7 ± 3.4^∗^	34.9 ± 7.4
Extracellular mass (kg)	25.8 ± 2.8	34.5 ± 2.0^∗^	30.7 ± 5.0^∗^	25.5 ± 2.5	33.8 ± 2.0^∗^	30.2 ± 4.7
Muscle mass (kg)	22.3 ± 2.6	36.6 ± 2.4	30.4 ± 7.7	22.7 ± 2.4	36.7 ± 2.4	30.6 ± 7.5
Total body potassium (g)	122.3 ± 11.4	190.3 ± 13.6	160.7 ± 36.6	121.6 ± 12.8	192.2 ± 13.7^∗^	161.5 ± 38.1
Dry weight (kg)	67.9 ± 10.8	83.4 ± 7.6	76.7 ± 11.9	66.3 ± 8.5	83.7 ± 7.9	76.1 ± 11.9
Protein (kg)	10.0 ± 1.2	14.7 ± 2.0	12.7 ± 2.9	10.2 ± 1.2	14.3 ± 1.6^∗^	12.5 ± 2.5
Mineral (kg)	4.1 ± 0.5	5.2 ± 0.6	4.7 ± 0.8	4.1 ± 0.5	5.0 ± 0.6^∗^	4.6 ± 0.7
Total body calcium (kg)	0.98 ± 0.13	1.50 ± 0.10	1.27 ± 0.29	1.00 ± 0.09	1.51 ± 0.10^∗^	1.29 ± 0.28^∗^
Glycogen (g)	483.8 ± 44.6	677.3 ± 40.6^∗^	593.2 ± 106.5^∗^	481.2 ± 48.5	672.8 ± 41.3	589.5 ± 106.5
Total body water (L)	39.1 ± 3.4	55.0 ± 4.1	48.1 ± 8.9^∗^	38.7 ± 3.9	54.7 ± 4.2^∗^	47.7 ± 9.0
Extracellular water (L)	17.3 ± 2.3	22.2 ± 1.3^∗^	20.1 ± 3.1	17.9 ± 1.7	21.6 ± 1.6	20.0 ± 2.5
Intracellular water (L)	21.8 ± 3.1	32.2 ± 3.1	27.7 ± 6.1	20.8 ± 2.4	33.1 ± 2.9^∗^	27.7 ± 6.8
Interstitial fluid (L)	12.8 ± 1.7	16.5 ± 1.0^∗^	14.9 ± 2.3	13.2 ± 1.3	16.0 ± 1.2	14.8 ± 1.8
Plasma fluid (L)	3.7 ± 0.5	4.7 ± 0.3^∗^	4.2 ± 0.7	3.8 ± 0.4	4.6 ± 0.3	4.2 ± 0.5
Extracellular solids (L)	4.9 ± 0.7	7.6 ± 0.5	6.5 ± 1.5	5.1 ± 0.5	7.7 ± 0.5^∗^	6.6 ± 1.4
Extracellular fluid (L)	18.3 ± 2.4	23.5 ± 1.5^∗^	21.2 ± 3.2	18.9 ± 1.8	22.9 ± 1.7	21.2 ± 2.6

*Note:* Values are presented as mean ± standard deviation.

^∗^Significant difference at *p* ≤ 0.05 for within-intervention comparisons between baseline and follow-up.

## Data Availability

The data used to support the findings of this study are available from the corresponding author upon reasonable request.

## Supporting Information

Supporting Information Supporting Table 1: Changes in raw bioelectrical parameters during the study. Values are expressed as mean ± SD.
