# Supplementary material for: The Impact of Short-Term Supplementation With Guanidinoacetic Acid and Creatine Versus Creatine Alone on Body Composition Indices in Healthy Men and Women: Creatine-Guanidinoacetic Acid Affects Body Composition
Source: J Nutr Metab. 2024 Oct 14;2024:7815807. doi: 10.1155/2024/7815807 (PMC11493483; doi:10.1155/2024/7815807)
Supplement: Supporting Information — Supporting Table 1: Changes in raw bioelectrical parameters during the study. Values are expressed as mean ± SD. [file 7815807.f1.docx]

**Supplementary Table 1.** Changes in raw bioelectrical parameters during the study. Values are mean ± SD.

|  | Baseline | Follow-up | |
| --- | --- | --- | --- |
|  |  | Creatine | Creatine-GAA |
| Impedance at 5 kHz (Ω)  Total  Men  Women | 538.7 ± 53.7  503.3 ± 29.1  593.8 ± 30.6 | 559.9 ± 147.3  556.4 ± 194.4  564.5 ± 51.3 | 522.0 ± 54.1  498.1 ± 45.9  555.4 ± 48.0 |
| Resistance 5 kHz (Ω)  Total  Men  Women | 538. ± 53.8  502.4 ± 29.2  593.3 ± 30.3 | 550.6 ± 109.9  539.8 ± 141.0  564.5 ± 51.3 | 521.4 ± 54.3  497.1 ± 45.8  555.4 ± 48/0 |
| Reactance 5 kHz (Ω)  Total  Men  Women | 32.8 ± 8.1  33.3 ± 9.4  32.0 ± 6.1 | 35.6 ± 9.1  40.6 ± 8.8  29.0 ± 3.7 | 32.9 ± 5.8  36.3 ± 4.1  28.2 ± 4.2 |
| Phase angle 5 kHz (Ω)  Total  Men  Women | 3.7 ± 0.6  4.0 ± 0.4  3.1 ± 0.5 | 3.8 ± 0.9  4.4 ± 0.7  3.0 ± 0.3 | 3.7 ± 0.7  4.2 ± 0.4  2.9 ± 0.4 |

|  | Baseline | Follow-up | |
| --- | --- | --- | --- |
|  |  | Creatine | Creatine-GAA |
| Impedance at 50 kHz (Ω)  Total  Men  Women | 425.0 ± 56.6  386.7 ± 30.5  484.4 ± 27.6 | 432.5 ± 76.7  399.5 ± 82.8  475.4 ± 40.4 | 418.0 ± 56.2  383.6 ± 37.5  466.2 ± 40.4 |
| Resistance 50 kHz (Ω)  Total  Men  Women | 402.3 ± 97.0  353.9 ± 95.0  477.6 ± 27.4 | 422.8 ± 70.1  387.5 ± 68.3  468.7 ± 40.2 | 411.0 ± 56.3  376.1 ± 36.9  459.8 ± 40.0 |
| Reactance 50 kHz (Ω)  Total  Men  Women | 78.6 ± 7.6  75.5 ± 6.9  83.3 ± 6.3 | 88.7 ± 42.9  94.0 ± 57.3  81.9 ± 5.7 | 78.3 ± 7.5  77.8 ± 7.4  79.0 ± 8.0 |
| Phase angle 50 kHz (Ω)  Total  Men  Women | 10.7 ± 0.9  11.3 ± 0.6  9.9 ± 0.6 | 11.7 ± 3.3  13.0 ± 3.9  9.9 ± 0.5 | 10.9 ± 1.2  11.7 ± 0.6  9.8 ± 0.7 |

|  | Baseline | Follow-up | |
| --- | --- | --- | --- |
|  |  | Creatine | Creatine-GAA |
| Impedance at 100 kHz (Ω)  Total  Men  Women | 388.7 ± 55.8  351.2 ± 30.4  447.0 ± 28.0 | 376.7 ± 75.6  326.9 ± 57.2  441.5 ± 37.1 | 384.3 ± 55.1  349.6 ± 35.9  432.9 ± 37.4 |
| Resistance 100 kHz (Ω)  Total  Men  Women | 380.6 ± 55.3  343.4 ± 29.8  438.4 ± 27.7 | 368.1 ± 76.4  318.2 ± 58.8  433.1 ± 36.8 | 376.4 ± 54.7  341.7 ± 35.3  425.0 ± 36.9 |
| Reactance 100 kHz (Ω)  Total  Men  Women | 80.4 ± 9.2  75.3 ± 6.9  88.4 ± 6.1 | 81.5 ± 9.5  76.7 ± 9.2  87.8 ± 5.5 | 80.2 ± 8.3  77.1 ± 7.6  84.5 ± 7.6 |
| Phase angle 100 kHz (Ω)  Total  Men  Women | 12.0 ± 0.8  12.4 ± 0.7  11.4 ± 0.6 | 12.9 ± 3.1  14.1 ± 3.8  11.5 ± 0.5 | 12.1 ± 1.0  12.7 ± 0.6  11.3 ± 0.7 |

|  | Baseline | Follow-up | |
| --- | --- | --- | --- |
|  |  | Creatine | Creatine-GAA |
| Impedance at 200 kHz (Ω)  Total  Men  Women | 356.9 ± 51.8  322.2 ± 27.5  410.9 ± 27.5 | 344.7 ± 68.4  298.3 ± 46.5  405.0 ± 37.5 | 350.6 ± 52.4  317.4 ± 33.1  397.0 ± 36.6 |
| Resistance 200 kHz (Ω)  Total  Men  Women | 346.9 ± 50.6  313.1 ± 26.9  399.6 ± 27.1 | 333.8 ± 68.3  287.9 ± 47.8  393.5 ± 37.1 | 340.8 ± 51.3  308.1 ± 32.0  386.4 ± 35.9 |
| Reactance 200 kHz (Ω)  Total  Men  Women | 85.5 ± 12.0  78.0 ± 7.4  97.3 ± 7.3 | 86.6 ± 12.8  78.4 ± 9.6  97.3 ± 7.1 | 84.4 ± 11.2  78.4 ± 8.6  92.8 ± 8.8 |
| Phase angle 5 kHz (Ω)  Total  Men  Women | 13.9 ± 0.7  14.0 ± 0.7  13.7 ± 0.7 | 14.9 ± 2.8  15.6 ± 3.6  13.9 ± 0.6 | 14.0 ± 0.8  14.3 ± 0.6  13.6 ± 0.8 |
